# Retrospective Analysis of Penicillin G Minimum Inhibitory Concentration for Gram-Positive Isolates of Non-Severe Clinical Mastitis

**DOI:** 10.3390/antibiotics14010021

**Published:** 2025-01-02

**Authors:** Stefanie Leimbach, Franziska Nankemann, Anne Tellen, Doris Klocke, Nicole Wente, Yanchao Zhang, Volker Krömker

**Affiliations:** Department of Microbiology, Faculty of Mechanical and Bioprocess Engineering, University of Applied Sciences and Arts, 30453 Hannover, Germany; stefanie.leimbach@hs-hannover.de (S.L.); franziska.nankemann@hs-hannover.de (F.N.); anne.tellen@hs-hannover.de (A.T.); doris.klocke@hs-hannover.de (D.K.); nicole.wente@hs-hannover.de (N.W.); yanchao.zhang@hs-hannover.de (Y.Z.)

**Keywords:** minimal inhibitory concentration, antimicrobial susceptibility, Gram-positive mastitis pathogens, procaine benzylpenicillin

## Abstract

**Background**: Despite penicillin having a longstanding reputation as being scientifically approved for the treatment of bovine mastitis, its market share and practical application rate seem rather low. While in some countries, cases of mild and moderate mastitis are treated almost completely with simple penicillin, in other countries, penicillin is rarely used as a mono-substance in udder tubes. **Methods**: Based on minimal inhibitory concentration (MIC) studies of 1489 isolates of Gram-positive microorganisms isolated from bovine mastitis cases, the extent to which penicillin preparations can fulfil their role as first-line treatment and in how many cases insufficient efficacy must be assumed was assessed in comparison with more recent studies on the achievable levels of active substances in milk. **Results**: Of the isolates, 76% had an MIC of ≤0.125 µg/mL and 95% of the isolates had an MIC of ≤1 µg/mL. **Conclusions**: The data show that in Northern Germany, it can be assumed that penicillin is a good choice in most cases of mastitis caused by Gram-positive mastitis pathogens, at least from the perspective of antibiotic resistance.

## 1. Introduction

A common practice in recent years has been to treat all cases of clinical mastitis with antimicrobial agents. Nowadays, however, antibiotic treatment is performed only for cases of mastitis that are likely to benefit. Evidence-based treatment approaches now recommend that mild and moderate (non-severe) mastitis caused by Gram-positive pathogens in animals without a long history of mastitis should be treated with antibiotics [1,2,3,4]. Antibiotic therapy is not recommended for cases with no pathogen growth or when non-severe clinical cases of Gram-negative pathogens like *E. coli* are detected, as these infections generally have a high tendency to self-cure [5,6]. Additionally, animals with a long history of mastitis or persistently elevated somatic cell counts are considered unworthy of treatment.

In Scandinavian countries, mastitis has predominantly been treated with narrow penicillins like benzathine benzylpenicillin (penicillin G) for many years. However, this practice is viewed sceptically by farmers and veterinarians in many other countries due to concerns about its limited spectrum of action, long withdrawal periods for milk, and the expected antimicrobial resistance of many mastitis pathogens [7]. Further hesitancy regarding the use of penicillin for mastitis remains due to limited clinical studies comparing its efficacy and the absence of mastitis-specific breakpoints for penicillin G. The Clinical and Laboratory Standards Institute (CLSI) performance standards provide breakpoints for the combination of penicillin-novobiocin for *S. aureus* and for different streptococci in bovine mastitis [8], but this antibiotic combination is not commonly used in Europe.

The use of in vitro susceptibility testing has not been the preferred practice to guide intramammary mastitis treatment [9]. However, susceptibility testing is crucial to identify the sensitivity of bacterial strains to specific antibiotics. Monitoring antimicrobial resistance aligns with the One Health concept brought forth by the World Health Organization (WHO) and the European Medicines Agency (EMA), which emphasise the importance of antibiotic stewardship. This involves ranking antibiotics according to their significance for human medicine. In other words, narrow-spectrum penicillins such as procaine benzylpenicillin are classified as category D (prudent) active substances and highly important antibiotics by the WHO and EMA, making them suitable as first-line treatments in animals wherever possible [10]. For mastitis therapy, this means that local treatment with narrow beta-lactam antibiotics is recommended.

Given this context, we hypothesise that susceptibility results vary according to location due to multiple factors, such as the animal’s response to treatment against a specific mastitis-causing pathogen. Therefore, we conducted a retrospective and observational analysis of penicillin G MICs for common pathogens in subclinical and non-severe clinical cases on dairy farms in Northern Germany.

This study aimed to examine whether the described reservations against the use of simple penicillin are based on high MIC values of the relevant mastitis isolates. For this purpose, MIC tests against penicillin were carried out on mastitis pathogens from Northern Germany. The pathogens tested were those known to be successfully combated with antibiotic therapy, i.e., Gram-positive pathogens.

## 2. Results

The MIC90 and MIC50 values obtained in this study are shown in Table 1. A total of 63% of all isolates were at the lowest MIC level. The lowest MIC50/90 values were detected for *C. bovis*, *S. simulans*, and *Str. dysgalactiae* (≤0.06 µg/mL, MIC50 and MIC90 were identical). The distribution pattern of *Str. agalactiae* was very dense, extending only over two dilution levels (≤0.06 µg/mL as MIC50 and 0.125 µg/mL as MIC90).

*S. aureus* and *S. haemolyticus* had the widest distribution patterns following *S. epidermidis*, ranging from ≤0.06 µg/mL to 16 µg/mL, and ≤0.06 µg/mL to 32 µg/mL, respectively. Out of the 296 *S. aureus* isolates, 222 were inhibited at penicillin concentrations of ≤0.06 µg/mL (MIC50), while the MIC90 value was 1 µg/mL.

Only two of the investigated species showed MIC50 values above 0.125 µg/mL—*S. succinus* (0.25 µg/mL) and *L. garviae* (0.5 µg/mL). MIC90 values varied depending on the pathogen species. An MIC90 of 0.125 µg/mL was found for *Str. agalactiae*, 0.25 µg/mL for *Str. uberis*, 0.5 µg/mL for *S. haemolyticus*, and *S. succinus*, 1 µg/mL for *S. aureus*, *L. garviae*, and *S. epidermidis*, and 4 µg/mL for *S. chromogenes*.

Indications of the presence of non-wild types that have acquired resistance are provided, in particular by the data for *Staphylococcus* species. These are most clearly recognisable in species that belong to the non-*aureus* staphylococci (NAS) and are found as a part of the normal skin flora of the teats.

For further *Corynebacterium* spp., *Enterococcus* spp. and *Staphylococcus* spp., less than 25 isolates were determined for each species, so the results were summarised at the genus level. The 296 isolates determined as *Staphylococcus* spp. showed an MIC50 of ≤0.06 µg/mL and an MIC90 of 1 µg/mL. An MIC50 of ≤0.06 and 0.125 µg/mL and an MIC90 of 0.125 and 0.25 µg/mL were determined for *Corynebacterium* spp. and *Enterococcus* spp., respectively.

## 3. Discussion

In recent years, the approach to treating bovine mastitis has shifted towards more selective antibiotic use, particularly for cases involving Gram-positive pathogens. Procaine penicillin, a narrow-spectrum antibiotic substance, has been recommended as a first-line treatment in such cases [10,11]. However, concerns about its limited pathogen spectrum, potential resistance, and economic disadvantages, such as extended milk withdrawal periods, have led to its cautious use in many regions. The aim of this study was to examine whether these reservations exist due to elevated MIC values and are, therefore, justified. Since modern selective therapy concepts limit the intramammary application of antibiotics to cases caused by Gram-positive pathogens, especially streptococci and staphylococci, the isolates investigated in this study were selected correspondingly.

Comparing the MIC results of this study to former studies, most values are in accordance or show little deviance. Bolte and colleagues [12], who also investigated mastitis pathogens isolated in German dairy farms, described identical MIC50 and MIC90 values for *Str. agalactiae* (MIC50 ≤ 0.06 µg/mL, MIC90 0.125 µg/mL) and *Str. dysgalactiae* (MIC50 and 90 ≤ 0.06 µg/mL). For *Str. uberis*, the MIC90 is also identical, while the MIC50 is higher in the study by Bolte and colleagues (0.125 compared to ≤0.06 µg/mL). The MIC50 for *S. aureus* is identical (≤0.06 µg/mL), whereas the MIC90 in our study is higher (1 µg/mL compared to 0.5 in Bolte and colleagues) [12]. These deviances do not exceed one dilution step and may be due to the number of investigated isolates. Further studies investigating MICs for penicillin against mastitis pathogens in other countries, such as Canada or the USA, showed similar results with small variations, possibly due to the different groupings of species or genera [13,14]. Similar to our study, MIC90 values published by these authors do not exceed 1 µg/mL, except for *Enterococcus* spp., for which Cameron and colleagues describe a value of 2 µg/mL [14].

In our study, the only species with an MIC90 above 1 µg/mL is *S. chromogenes* (4 µg/mL). Since NAS species are physiological commensals, which can be isolated from the skin of teats and udders and do not seem to carry relevant pathogenicity factors, they are usually described as minor mastitis pathogens [15]. Therefore, in terms of antibiotic therapy, it may be reasonable to focus mainly on the major pathogens such as *Str. uberis* and *S. aureus*.

Regarding all investigated isolates, it can be summarised that 95% of these 1489 isolates showed an MIC90 of 1 µg/mL or lower. This information is very useful for estimating the possible success of a sole penicillin-based therapy. However, as no mastitis-specific MIC breakpoints for penicillin exist, and available breakpoints derived from other sources are lower than 1 µg/mL, it is necessary to regard further pharmacologic factors such as the distribution of the substance within the udder tissue and milk, and the respective concentrations that are maintained during therapy.

Ehinger and Kietzmann (2000) showed, in their studies on isolated perfused bovine udders, that almost all locally applied penicillin preparations investigated exceeded the MIC against relevant mastitis pathogens in all localisations in the glandular tissue [16].

In a more recent study with dairy cows with daily milk yields over 30 L, concentrations of 2459.06, 2648.71 and 4486.51 ng/mL milk (geo. mean) were determined 24 h after intramammary administration of 200,000 IU, 300,000 IU or 600,000 IU benzylpenicillin in the form of an oily suspension [17]. After a single application, the level in the milk remained above 125 ng/mL for 35 h for all three preparations.

For time-dependent antibiotics such as benzylpenicillin, maximum kill in vitro is achieved at three to four times the MIC, and the most important predictor for elimination of infection is the time above the MIC [18].

On the German market, the lowest-dose preparation has a dosage of 600,000 IU per tube. If the aforementioned rules are taken into account, very good efficacy up to an MIC of the bacterial isolates of 1.5 µg/mL can be assumed. Thus, unless proven otherwise, penicillin could and should be used as a prudent first-line intramammary antibiotic treatment for clinical bovine mastitis cases, as it has already been successfully implemented in other Northern European countries such as Denmark. However, it would be of great benefit to be able to reliably identify the cases that are caused by penicillin-resistant bacteria and need to be provided with more suitable antibiotic substances. Some rapid test systems addressing this issue are already available and could be implemented in modern mastitis treatment concepts.

Extrapolating laboratory-determined MIC values to clinical outcomes in farm animals with mastitis has limitations. Laboratory conditions do not fully replicate the udder’s complex environment, where factors like the pharmacokinetics and pharmacodynamics of antibiotics in vivo, variability in immune response among animals, and differences in the physiological conditions within the mammary can affect treatment efficacy. Antibiotic distribution in udder tissue further influences outcomes. These variables underscore the need for caution when directly applying MIC findings to predict treatment success in the field, and future studies integrating in vivo data are essential to validate their clinical relevance.

## 4. Materials and Methods

### 4.1. Isolation and Identification of Pathogens

The isolates used in this study were randomly chosen from the strain collection of the University of Applied Sciences and Arts in Hannover, Germany. The examined mastitis pathogens were obtained from subclinical and clinical mastitis cases in dairy cows from 89 farms in Northern Germany that were sent in as part of routine diagnostics over the last seven years (since 2017). The herd sizes varied between 50 and 2000 cows and had an annual average milk yield of between 6500 and 14,000 kg ECM and bulk tank somatic cell contents of between 70,000 and 380,000 cells/mL. The milk samples sent in contain no further information but are mainly clinical cases of mastitis (>80%). Furthermore, no data are available on the respective lactation stages of the occurrence of the cases or on first or recurrent cases. Due to the herd-specific effects and uneven distribution of the species between the farms and the diversity of the isolates, an investigation of the temporal dynamics in the relatively short study period of 7 years is not meaningful.

Milk samples were collected, and pathogens were identified following the German Veterinary Medical Association (GVA) guidelines [19]. Each milk sample was plated onto a quadrant of esculin blood agar (Oxoid, Wesel, Germany) and incubated at 37 °C. After 24 and 48 h, the resulting colonies were subjected to microbiological examination. Colony morphology, haemolysis patterns, and esculin hydrolysis were assessed for each blood agar plate. Further differentiation of pathogens involved Gram staining and biochemical tests, including catalase activity assays (3% H_2_O_2_; Merck, Germany) and a clumping factor test (DiaMondiaL Staph Plus Kit, Sekisui Virotech, Germany) for the distinction between *S. aureus* and NAS. Following Saiful and colleagues [20], *S. aureus* isolates were confirmed by detecting the specific nuc gene. For the identification of esculin-negative streptococci according to Lancefield groups C and B (like *Str. dysgalactiae* or *Str. agalactiae*), serological tests (DiaMondiaL Streptococcal Extraction Kit Sekisui Virotech, Germany) were used. A distinction between *Str. uberis* and *Enterococcus* species was performed using a Rambach agar modified by Watts and colleagues [21].

For identifying Gram-negative rods, cytochrome oxidase C activity (Bactident oxidase, Merck, Germany) and an oxidative-fermentative test (OF basal medium with added D(+)-glucose-monohydrate, Merck, Germany) were conducted. Coliform bacteria capable of fermenting glucose were further identified using Chromocult coliform agar (Merck, Germany), with *Escherichia coli* appearing as blue colonies after incubation for 24 h at 37 °C. Pink colonies on the Chromocult agar were subjected to additional analysis, with *Klebsiella* species differentiated from other coliform bacteria based on their lack of mobility in the oxidative fermentative test.

Afterwards, the bacteriological results were confirmed using the matrix-assisted laser desorption time-of-flight mass spectrometry (MALDI-TOF, Bruker Daltonics GmbH & Co. KG, Bremen, Germany). A cut-off value of ≥2.0 was used for the identification at the species level, and a value of ≥1.7 was used for identification at the genus level.

A total of 1489 isolates were included in this study. These comprised 30 *Corynebacterium (C.) bovis*, 26 *Lactococcus (L.) garviae*, 296 *Staphylococcus (S.) aureus*, 52 *S. chromogenes*, 46 *S. epidermidis*, 83 *S. haemolyticus*, 69 *S. simulans*, 26 *S. succinus*, 70 *Streptococcus (Str.) dysgalactiae*, 51 *Str. agalactiae* and *425 Str. uberis isolates*. In addition, 11 isolates from *Corynebacterium* spp. other than *C. bovis*, 8 *Enterococcus* spp., and 296 *Staphylococcus* spp. (other than the aforementioned species) were found. Because fewer than 25 isolates per mastitis species were determined for each of these species, they were grouped and analyzed at the genus level.

### 4.2. Antimicrobial Susceptibility Testing

Antimicrobial susceptibility testing was conducted using the broth microdilution method in accordance with the DIN 58940/ISO20776-1:2020 protocol [22]. The MIC of mastitis pathogens was determined for penicillin G. Therefore, the sterile polystyrene microtiter plates (Greiner Bio One, Germany) contained antimicrobial concentrations in a two-fold dilution series. To investigate *Streptococcus* spp., 5% of defibrinated horse blood (Oxoid) was added. The microorganisms were tested against penicillin G in concentration levels ranging from 0.06 to 32 µg/mL. The reference strains used were *S. aureus* ATCC 29213 and *E. coli* ATCC 25922 (Leibniz Institute DSMZ—German Collection of Microorganisms and Cell Cultures, Braunschweig, Germany).

In each well containing 100 µL of the respective antimicrobial dilution levels, 5 µL of an immediately prepared bacterial solution was added to a final bacterial solution level of 5 × 10^5^ cfu/mL. Wells filled with Mueller–Hinton–Bouillon represented the positive controls and were also incubated. Wells containing only liquid nutrient medium without adding bacterial solution represented the negative control. The inoculated microtiter plates were incubated at 37 °C for 24 h. In the evaluation, the turbidity of the respective well after the incubation period was designated as bacterial growth.

If >25 isolates of one mastitis species were determined, the results were reported at the species level. For further species, the results were given at the genus level. Both the distribution of the individual isolates according to the MIC values determined and the MIC50 and MIC90 values are given. The MIC50 and MIC90 values are defined as the lowest concentrations at which the multiplication of at least 50% and 90% of the tested bacteria is inhibited [22].

## 5. Conclusions

Our results indicate that up to 95% of the 1489 isolates were susceptible to penicillin G in vitro. Considering the particularities of this study, penicillin G is likely as effective as other beta-lactam antibiotics, making them suitable as first-line treatments for animals wherever possible. However, extrapolating laboratory-determined MIC values to clinical outcomes in mastitis is limited by differences between controlled conditions and the complex in vivo environment, highlighting the need for caution and further in vivo validation studies.

## Figures and Tables

**Table 1:** Distribution of the minimal inhibitory concentration (MIC) for penicillin G of different mastitis species (n = 1174) and genera (n = 315) * isolated from Northern German dairy farms ^1^.

		Percent of Isolates at Each Indicated MIC (µg/mL)										
Species	n	≤0.06	0.125	0.25	0.5	1	2	4	8	16	32	>32
*C.* ^2^ *bovis*	30	**93.3**	-	3.3	3.3	-	-	-	-	-	-	-
*L.* ^3^ *garviae*	26	15.4	3.8	7.7	**61.5**	7.7	3.8	-	-	-	-	-
*S.* ^4^ *aureus*	296	**75**	6.1	4.7	3	4.7	3.4	1.4	1	0.7	-	-
*S. chromogenes*	52	**75**	5.8	-	5.8	-	1.9	9.6	1.9	-	-	-
*S. epidermidis*	46	47.8	**8.7**	19.6	10.9	8.7	-	-	-	2.2	2.2	
*S. haemolyticus*	83	44.6	**10.8**	21.7	13.3	7.2	-	-	-	2.4		
*S. simulans*	69	**94.2**	-	2.9	-	-	1.4	1.4	-	-	-	-
*S. succinus*	26	-	7.7	**69.2**	19.2	3.8	-	-	-	-	-	-
*Str.* ^5^ *dysgalactiae*	70	**94.3**	2.9	1.4	1.4	-	-	*-*	-	-	-	-
*Str. agalactiae*	51	**70.6**	29.4	-	-	-	-	*-*	-	-	-	-
*Str. uberis*	425	**45.2**	31.1	18.6	3.3	1.4	0.2	0.2	-	-	-	-
*Corynebacterium* spp.*	11	**72.7**	18.2	-	9.1	-	-	*-*	-	-	-	-
*Enterococcus* spp.*	8	37.5	**12.5**	12.5	-	-	-	37.5	-	-	-	-
*Staphylococci* spp.*	296	**75**	6.1	4.7	3.0	4.7	3.4	1.4	1.0	0.7	-	-

^1^ MIC50 are displayed in **bold** and MIC90 are displayed underlined, respectively. Bold and underlined digits indicate that MIC50 and MIC90 were identical. ^2^ *Corynebacterium*, ^3^ *Lactococcus*, ^4^ *Staphylococcus*, ^5^ *Streptococcus*. * cases without species identification.

## Data Availability

The original contributions presented in this study are included in the article. Further inquiries can be directed to the corresponding author.
